# The impact of sustentaculum tali fracture on clinical outcome in patients affected by isolated calcaneal fractures

**DOI:** 10.1007/s00590-023-03760-2

**Published:** 2023-10-19

**Authors:** Giacomo Sani, Niccolò Giabbani, Luigi Zanna, Roberto Buzzi, Angelica Sofia Pio, Vieri Rastrelli, Cosimo Nardi

**Affiliations:** 1 Department of Surgery and Translation Medicine, University of Florence - Azienda Ospedaliero-Universitaria Careggi, Largo Palagi 1, 50134 Florence, Italy; 2 Department of Experimental and Clinical Biomedical Sciences, Radiodiagnostic Unit N. 2., University of Florence - Azienda Ospedaliero-Universitaria Careggi, Largo Palagi 1, 50134 Florence, Italy

**Keywords:** Calcaneus, Fracture, Reduction, Internal fixation, Subtalar joint, Sanders, Sustentaculum tali, Calcaneocuboid joint, Clinical outcome

## Abstract

**Introduction:**

The sustentaculum tali is displaced in almost half of calcaneal fractures and during surgical fixation represents one of the main reference points upon which the other bone has to be reduced. The purpose of this study was to investigate which subtalar joint fracture pattern is more frequently associated with sustentaculum tali involvement. Furthermore, correlation between postoperative clinical outcome and sustentaculum tali integrity was performed.

**Material and methods:**

Patients with isolated calcaneal fractures were analyzed. Sanders-type fracture and involvement of both sustentaculum tali and calcaneocuboid joint were detected on computed tomography imaging; postoperative AOFAS scores were analyzed according to sustentacular involvement.

**Results:**

Fifty calcaneus fractures in 47 patients were included in the final analysis. The sustentaculum tali was fractured in 18 cases (36.0%), thus contradicting its supposed constant position. Sanders type 3 and 4 fractures were more frequently associated with fractured sustentaculum than type 2 (*p* = 0.012). Sanders type 4 fractures were associated with displaced sustentacular fragment significantly more than type 2 and 3 (*p* = 0.043). Patients with intact sustentaculum tali reported significantly higher (*p* < 0.001) mean AOFAS scores than the uninjured group (84.4 ± 9.1 and 74.3 ± 9.5, respectively).

**Conclusion:**

Sanders type 3 and 4 fractures were more frequently associated with sustentaculum tali and/or calcaneocuboid joint involvement than simpler fractures. Injury of sustentaculum tali was related to significant worse postoperative clinical outcomes, underlying the relevance of this fragment on clinical course.

## Introduction

Calcaneal fractures represent the 2% of all fractures, affecting young to middle-aged patients, especially men during working age [1]. The traumatic mechanism underlying calcaneal fractures is axial loading, which occurs during a fall from a height or a traffic accident [2]. Three-quarters of calcaneal fractures are intra-articular, involving the posterior subtalar joint (STJ) [3, 4]. Open reduction and internal fixation is the treatment of choice for displaced intra-articular calcaneal fractures even though comparative studies between operative treatment and nonoperative care showed comparable results in the literature [5]. The surgical management should be individualized, and operative treatment has to be reserved for selected cases with certain patient factors and fracture patterns [5]. Surgical treatment aims to restore calcaneal anatomy and subtalar motion; however, many complications such as post-traumatic osteoarthritis, chronic impingement of the flexor hallucis longus tendon, varus of the hindfoot, persistent pain of the medial hindfoot and wound dehiscence may occur [6]. Radiographs are the first-level imaging procedures to investigate calcaneal fractures, even though significant limitations, due to the two-dimensional imaging of three-dimensional structures, superimposition, geometric distortion, and anatomic noise, cannot be solved [7]. Computed Tomography (CT) imaging allows to evaluate the calcaneus bone in all reconstruction planes, to determine the fracture patterns according to Sanders classification [8], and to perform an accurate preoperative planning in order to achieve adequate surgical treatments [9].The sustentaculum tali is the bone portion of greatest interest among surgeons, and it is often referred to as the constant fragment due to its constant relationship with the talus [10], 10. During surgical fixation the sustentaculum tali represents one of the main reference points upon which the other bone has usually to be reduced on, in order to restore the congruity of the subtalar joint [4, 5, 8, 10, 11].

Accordingly, preoperative CT scan is mandatory in all calcaneal fractures to carry out correct and reliable surgical planning. In the literature [10, 11], the sustentacular fragment can be accurately considered as constant and can be consistently relied on to maintain anatomic alignment; however, postoperative clinical outcome related to specific type of fracture was not investigated.

This retrospective study aimed to investigate both which STJ pattern is more frequently associated with sustentaculum tali involvement, and the correlation between Sanders-type fracture and integrity of the Calcaneocuboid Joint (CCJ). The second endpoint was to correlate the postoperative clinical outcome of calcaneal fractures with sustentacular involvement compared to fractures with intact sustentaculum. We hypothesized that the sustentaculum tali is more frequently involved in Sanders type 3 and 4 fractures, and that postoperative clinical outcomes of fractures with intact sustentaculum are more favorable than those with fractured sustentaculum tali.

## Materials and methods

We analyzed all patients surgically treated for calcaneal fractures by a single experienced surgeon between December 2018 and December 2020 in our trauma center. Inclusion criteria were represented by isolated calcaneal fractures, open reduction and internal fixation through lateral approach, and postoperative follow-up more than 1 year. Patients with fractures of other tarsal bones, malleolar fractures, open fractures, follow-up less than 1 year and patients who underwent nonoperative treatment or minimally invasive surgical procedures, were excluded.

All patients were admitted to our emergency department, and both clinical and radiological examinations were assessed. Preoperative radiographs and CT scans were carried out on all patients.

### CT evaluation of bone structures

**Fig. 1:**
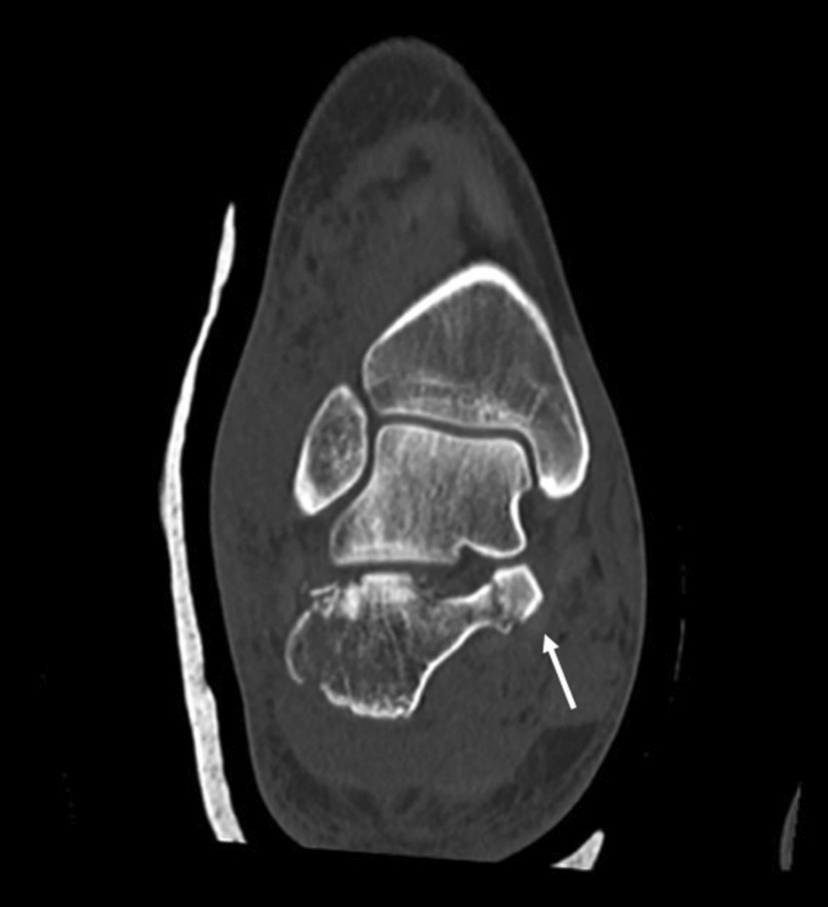
Semicoronal CT section. Fracture of the calcaneus. The white arrow shows the extension of the fracture line to the body of the sustentaculum tali

**Fig. 2:**
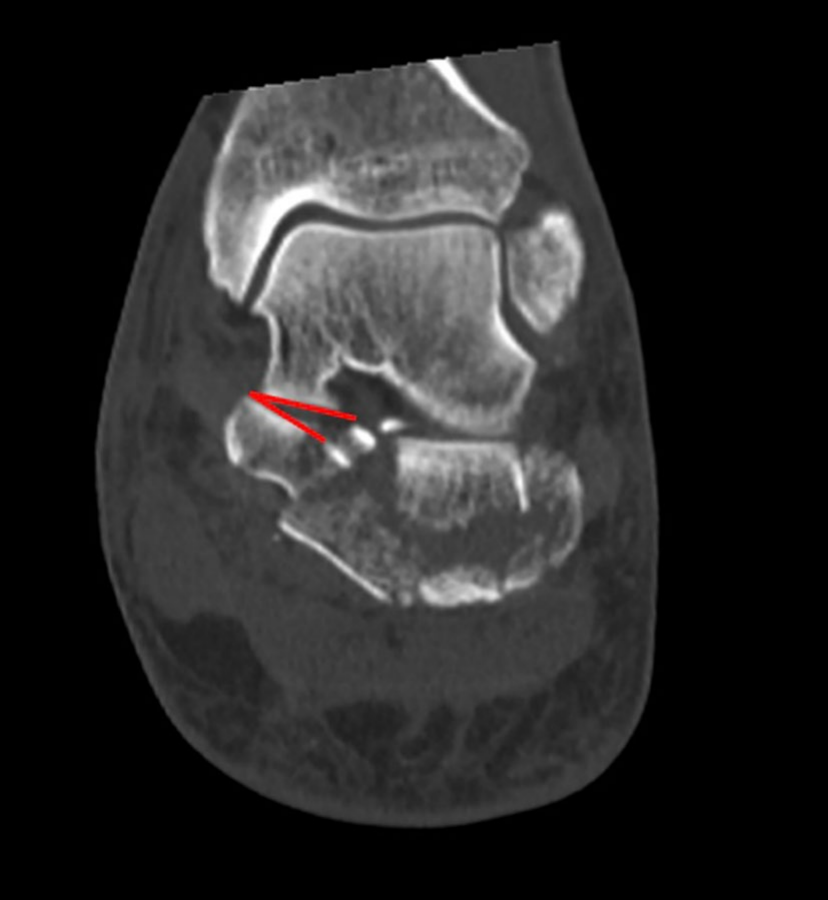
Semicoronal CT section. Fracture of the calcaneus involvement of the sustentacular fragment. Note that the sustentacular fragment is both tilted and fractured. The angle between the talar and calcaneal facets of the middle subtalar joint is 27°

Radiographs were performed with Polydoros LX 30 X-ray generator (Siemens, Erlangen, Germany) and included anteroposterior, lateral, and internal oblique views of foot and an axial view of calcaneus. CT scan was performed using a helical scanner with 128 detector rows CT (Philips Brilliance iCT Medical Systems, Cleveland, OH, USA) with the following parameters: tube voltage 140 kV, pixel size 0.465 mm, field of view 121 mm, thickness and reconstruction intervals 1 mm, current × exposure time 179 mAs, rotation time 0.4 s, beam collimation 128 × 0.67 mm, spacing 0.6 mm, and reconstruction core 131 s/3. CT images were obtained by reconstructions on axial (parallel to the sole of the foot), semi-coronal (perpendicular to the STJ), and sagittal (perpendicular to the sole of the foot) sections. Semi-coronal slices were examined to detect the position and number of fracture lines through the posterior STJ. Fractures were classified as 2, 3, or 4 part according to the number of fracture lines and as A, B, or C according to the lateral, central, or medial position (Sanders classification) [12].The involvement of the sustentacular fragments was evaluated on axial and semi-coronal sections. The sustentaculum tali was regarded as involved in case of a fracture line involving its body and/or in case of extension of STJ fracture line to the sustentacular fragment. Therefore, fractures were divided into two main groups according to the involvement of the sustentacular fragment, namely intact sustentaculum tali (Group A) and involved sustentaculum tali (Group B) (Fig. 1). Angulation and translation of the sustentacular fragment were calculated in group B fractures. The angulation of the sustentacular fragment was calculated as the angle between the talar and calcaneal facets of the middle subtalar joint (Fig. 2). The sustentaculum tali was defined as displaced if the angle between the talar and calcaneal facets of the middle STJ was > 5° on the semi-coronal and/or sagittal sections or if the translation of the fracture was equal to or greater than 2 mm [11]. The sustentaculum tali was considered un-displaced in all other cases. Axial and sagittal sections were crucial for the evaluation of CCJ involvement. Such fractures were classified as un-displaced or displaced (simple and multi-fragmentary). The relationship among the specific type of Sanders fracture, sustentacular fragment, and CCJ involvement was analyzed.

### Surgical procedure

**Fig. 3:**
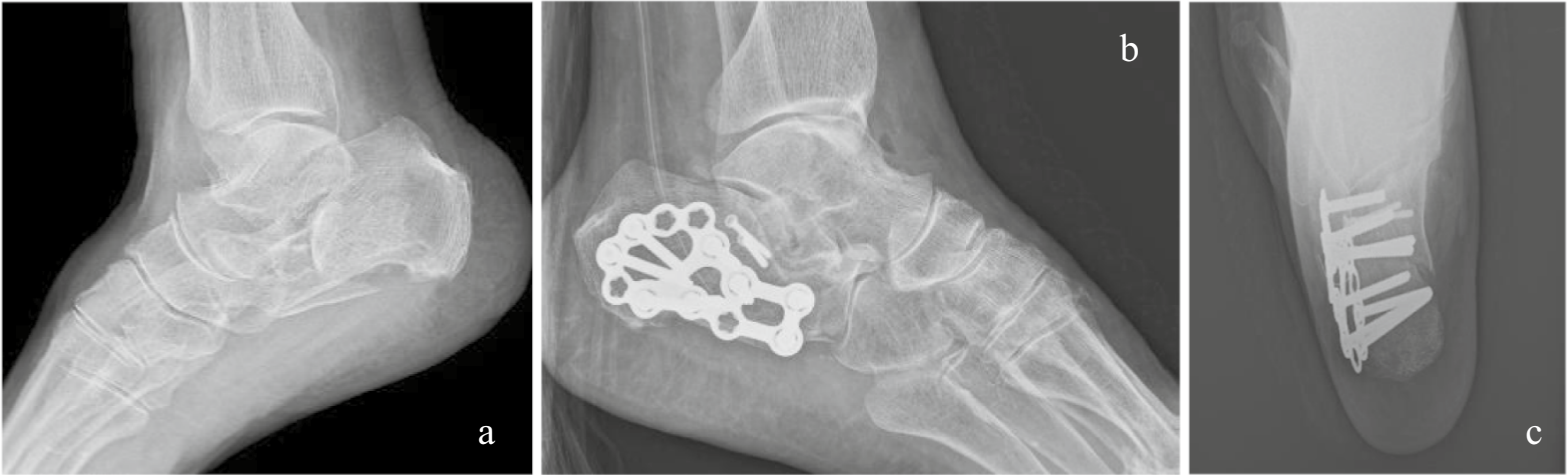
**a** Radiograph of a comminuted fracture of the calcaneus. **b** Lateral view and **c** axial view Harris of postoperative fracture of the calcaneus. Reduction and synthesis performed with lateral plate and screws and sustentaculum tali screw

All patients underwent open reduction and internal fixation through extended lateral approach on the seventh day on average (range 3–12 days) after resolution of post-traumatic swelling was solved. After spinal anesthesia patients were placed in a supine position with a pillow beneath their affected foot and a thigh tourniquet was positioned. The lateral *L*-shaped skin was performed directly to the bone to create thick soft tissue flaps. Bone fragments and the amount of joint involvement were evaluated. Particular attention was paid to the assessment of the sustentaculum tali. When sustentaculum tali was fractured and displaced, even minimally, it was managed through the lateral approach. The approach was performed through an incision that begins laterally 3–4 cm proximal to the calcaneal tuberosity and 1–2 cm anterior to the heel cord, extended distally and continued retrofibularly to the junction of the dorsal and plantar skin, where a smooth curve was made, curving the incision anteriorly toward the calcaneocuboid joint and the fifth metatarsal base. The fracture line at the level of the Gissane angle was identified, and the thin lateral wall was retracted inferiorly to expose the articular fracture fragments. Then, bone fragments were fixed temporarily with *k* wires. Intraoperative fluoroscopy was routinely employed in our institute to evaluate the quality of the reduction achieved, determine the presence of intra-articular osteochondral bodies, and confirm extra-articular placements of the screws. Fixation was subsequently performed using Smith and Nephew locking calcaneal plates (Fig. 3). Attention was turned to restoring the height, width, and length of the calcaneus, which was accomplished by complete reduction of articular fragments. In such cases when the sustentaculum tali was displaced and the lateral approach was not sufficient to achieve satisfactory reduction, an additional medial approach was performed. The medial approach was made through a horizontal incision about 5 cm long and centered over the palpable sustentaculum approximately 2 cm below and 1 cm in front of the medial malleolus and behind the tuberosity of the navicular. After opening the deep fascia, the posterior tibial tendon (PT) sheath was incised, flexor digitorum longus (FDL) and the PT retracted dorsally, the neurovascular bundle, plantar and posterior to the approach, was retracted plantarly. The interval between the PT and the PTA allowed to easily approach the inferior cortical exit point of the fracture. Fixation was typically accomplished with 1 or 2 cannulated screw or mini-plates as necessary. All the patients routinely received standard perioperative antibiotic prophylaxis. Anti-thromboembolic prophylaxis consisted of low molecular weight heparin and was administered until the patient regained full weight-bearing gait.

### Clinical outcomes

Clinical and imaging assessments were performed at 1, 2, 3, 6, 12 months postoperatively and at the final follow-up (> 1 year). Postoperatively, the foot was immobilized in a splint for 3 weeks; then, toe-touch weight bearing was permitted for 3 weeks with a walking boot. After 6 to 8 weeks and proper radiographic follow-up, full weight bearing without the walking boot was allowed. The American Orthopedic Foot and Ankle Society (AOFAS) score of the hindfoot was used to evaluate the clinical outcome of patients at the final follow-up, at least 1 year after surgery. The AOFAS score is one of the most widely scoring systems used to assess the functional ability and physical examination. Such score ranges from 0 to 100, with 100 being the maximum indicating no pain and full function. AOFAS score has been widely adopted over the years until it becomes the reference standard for the evaluation of patients after foot and ankle surgery. Based on the AOFAS hindfoot score, the result was considered excellent, good, fair, and poor when the total score ranged from 100 to 90, 89 to 75, 74 to 50, and below 50 points, respectively [13].

### Statistical analysis

All CT examinations were independently reviewed by two radiologists with 9 and 5 years of musculoskeletal imaging experience, respectively. Inter-reader reliability for the measurements was calculated using the Cohen kappa coefficient. Kappa values of 0.01–0.20, 0.21–0.40, 0.41–0.60, 0.61–0.80, 0.81–0.99, and 1 represented slight, moderate, substantial, near-perfect, and perfect agreement, respectively. Finally, the Chi-square test was used to examine differences between groups. Continuous variables were compared using paired and unpaired *t* test as appropriate. The significance threshold was set at a *p *value < 0.05. All statistical analyses were performed using GraphPad Prism 7.0 (GraphPad Software, Inc., San Diego, CA).

## Results

**Fig. 4:**
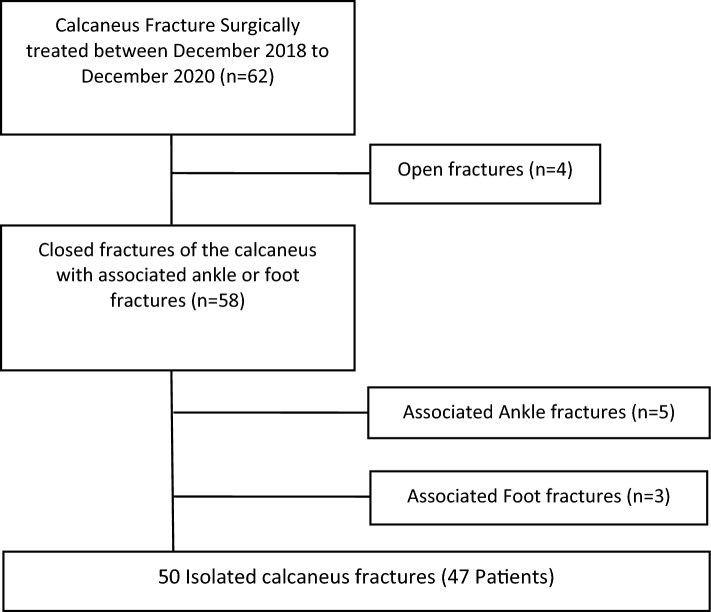
Flowchart of the selection criteria for enrolling patients

According to inclusion and exclusion criteria, 50 fractures (47 patients) were included in the final analysis (Fig. 4). The mechanisms of injury were road traffic accidents in 11 cases and fall from a height in 38 cases, whereas in 1 case it was not known. The patients were 34 men (72.0%) and 13 women (28.0%) with a mean age of 46 years (range 24–78). The right side was affected in 24 cases (48.0%) and the left side in 20 (40.0%). Three patients had bilateral fractures (12.0%).

### Fractures’ classification and sustentaculum tali involvement

The fractures’ classification according to Sanders showed that 2-part fractures were the most common (31 cases, 62.0%), whereas more complex fracture types including 3-part and 4-part fractures were less easily found (Table 1). The sustentaculum tali was intact in 32 cases (64.0%, group A) and fractured in 18 cases (36.0%, group B). In group B, the sustentacular fragment was un-displaced in 10 fractures and displaced in 8 fractures (Table 2). Sanders type 3 and 4 fractures were significantly (*p* = 0.012) more frequently associated with fractured sustentacular fragment (11 out of 19, 57.9%) than type 2 fractures (7 out of 31, 22.6%) (Table 3). Furthermore, Sanders type 4 fractures (5 out of 6, 83.3%) were significant associated with displaced sustentacular fragments more often than both type 2 (1 out of 7, 14.3%) and 3 fractures (2 out of 5, 40.0%) (*p* = 0.043) (Table 4).

**Table 1:** Classification of the fractures according to Sanders

Sanders type	Fractures
Two-part fractures	31 (62%)
Three-part fractures	13 (26%)
Four-part fractures	6 (12%)

**Table 2:** Involvement of sustentaculum tali

Sustentaculum tali	Fractures
**Intact (group A)**	**32 (64%)**
**Involved (group B)**	**18 (36%)**
Undisplaced	10
Displaced	8

**Table 3:** Types of fractures according to Sanders classification and correlation with sustentacular involvement

Sanders type	Intact sust. tali	Involved sust. tali	*p* value
Two-part fractures	24 (77.4%)	7 (22.6%)	*0.012*
Three- and 4-part fractures	8 (42.1%)	11 (57.9%)	

Statistically significant value in italics *P*-values < 0.05

**Table 4:** Types of fractures according to Sanders classification and correlation with sustentacular involvement: undisplaced vs. displaced sustentaculum tali

Sanders type	Sust. tali involved and undisplaced	Sust. tali involved and displaced	*p* value
Two-part fractures	6 (85.7%)	1 (14.3%)	*0.043*
Three-part fractures	3 (60.0%)	2 (40.0%)	
Four-part fractures	1 (16.7%)	5 (83.3%)	

Statistically significant value in italics *P*-values < 0.05

### CCJ involvement

The involvement of the CCJ is shown in Table 5. CCJ was intact in 14 cases (28%) and fractured in 36 cases (72.0%). Among fractured cases, 9 were un-displaced, 11 were displaced with a simple fracture line, and 16 were multi-fragmentary. In Sanders type 3 and 4 fractures CCJ was affected in 76.9% and 83.3% of cases, respectively, whereas in Sanders type 2 fractures CCJ was involved in 67.7% of cases (Table 6), although without reaching a statistical significance (*p* = 0.661).

**Table 5:** Involvement of calcaneocuboid joint

Calcaneocuboid joint	Fractures
**Not fractured**	**14 (28%)**
**Fractured**	**36 (72%)**
Undisplaced	9
Displaced, simple	11
Displaced, multi-fragmentary	16

**Table 6:** Types of fractures according to Sanders classification and correlation with calcaneocuboid joint involvement

Sanders type	CCJ involved	CCJ intact	*p* value
Two part	21/31 (67.7%)	10/31 (32.3%)	*0.661*
Three part	10/13 (76.9%)	3/13 (23.1%)	
Four part	5/6 (83.3%)	1/6 (16.7%)	

Statistically significant value in italics *P*-values < 0.05

### AOFAS scores

AOFAS score data were collected on average at 17 months after surgery (range 13–22 months). Overall average AOFAS score for all fractures was 80.8 ± 10.6 (range 70–96). The mean AOFAS score of the group A, patients with intact sustentacular fragment, was 84.4 ± 9.1 (range 70–96). The clinical outcome of the group B, patients with involved sustentacular fragment (74.3 ± 9.5, ranging between 75 and 92), was significant lower than the group A (*p* < 0.001). Furthermore, in the group B, the patients with displaced sustentaculum had worse AOFAS score than patients with no displaced sustentaculum (mean values 71.1 ± 9.5 and 76.8 ± 9.3, respectively) (Table 7).

**Table 7:** Mean American Orthopedic Foot and Ankle Society (AOFAS) score concerning sustentaculum tali involvement

Sustentaculum tali	Mean AOFAS score	*p* value
**Intact**	**84.4 ± 9.1**	*< 0.001*
**Involved**	**74.3 ± 9.5**	
Undisplaced	76.8 ± 9.3	
Displaced	71.1 ± 9.5	

Statistically significant value in italics *P*-values < 0.05

### Reliability

Inter-reader reliability showed from substantial to perfect agreement for the assessment of Sanders classification (0.76), displacement of the sustentacular fragment (0.81), angulation of the sustentacular fragment (0.85), CCJ involvement (0.73), and sustentacular fragment width (1).

## Discussion

The evaluation of the posterior STJ, sustentaculum tali, and CCJ on CT imaging is crucial for calcaneal fractures’ treatment [8]. The current study showed that the sustentaculum tali was significantly more frequently fractured and/or displaced in Sanders-type fractures 3 and 4 than type 2, resulting to a less reliable reference point during surgical open reduction. Furthermore, the clinical outcomes of calcaneal fractures with intact sustentacular fragment were significantly better than those with involved sustentaculum (*p* < 0.001).

According to the AO principles [14], anatomic reduction and stable osteosynthesis are the basis for restoring the best function of the injured bone segments. In calcaneal fractures, emphasis is commonly placed on the posterior subtalar facet reconstruction, narrowing of the width of the calcaneus to prevent lateral impingement of the peroneal tendons, and re-establishment of the normal height of the calcaneus [15, 16]. Nevertheless, the middle and anterior subtalar facets, although smaller than the posterior subtalar facet, carry on proportionally greater loads than the posterior facet. A proper reduction of middle and anterior calcaneal process is indeed crucial to preserve the length of the lateral column; in fact, a short lateral column results in forefoot abductions and decreases the longitudinal arch restricting eversion movement and causing some degrees of varus of the calcaneus [17, 18]. CT imaging is a mandatory examination to investigate fractures in order to develop a reliable surgical planning, mainly focusing on posterior STJ and sustentaculum tali involvement. Sanders developed the most commonly used classification system, based on the number and location of STJ articular fracture fragments on semicoronal slices. Fractures were further subdivided into subtypes A, B, and C in relation to the location of the primary fracture line. Subtype-C fractures were described as those in which the primary fracture line was the most medial, near the base of the sustentaculum tali [8].The sustentaculum tali is mainly made of cortical bone and has an important function in supporting the middle subtalar facet. An anatomic review of fifty calcaneal specimens described multiple variations in length, confluence with the anterior facet, and width of the sustentaculum tali, even in not fractured calcaneus bones [19]. The sustentacular fragment is considered a reference point for the reconstruction of intra-articular calcaneal fractures [10, 20] because it normally maintains proper relationships with the talus through the interosseous talus-calcaneus ligament and deep fibers of the deltoid ligament. Because of these features, it is also called "constant fragment" [9], although studies have shown that this fragment is frequently fragmented affecting the accuracy of surgical reconstructions[10, 11]. Berberian et al. [10] analyzed 80 patients with 100 displaced intra-articular calcaneal fractures reporting angulation and translation of the sustentaculum fragment in 42 cases. In a similar study on 94 patients with calcaneal fractures, Gitajn et al. [11] reported an incidence of 44.3% for fractures with sustentaculum tali involved, most of which were undisplaced (76.6%). Our results were comparable with those of Berberian et al. [10] and Gitajn et al. [11]. Involvement of sustentaculum tali was found in 18 of 50 intra-articular calcaneal fractures (36%) contradicting its supposed anatomic constancy. Based on both the above-mentioned analysis and our findings we may state that the "constant fragment" does not appear to be always constant. In the present study the more fragmented was the fracture—according to Sanders, i.e., type 3 and 4—the more sustentaculum tali was involved and/or displaced. Therefore, we strongly claim that the sustentaculum tali should not be taken as the only reference point for surgical reduction, especially in multi-fragmentary fractures.

The clinical outcome of our cohort was good (mean AOFAS score 80.8 ± 10.6). In line with the literature, the surgical treatment was effective and good to excellent outcome may be achieved, especially if compared with the nonoperative treatment [21] [22]. The mean AOFAS score was 84.4 ± 9.1 in the group A—patients with intact sustentacular fragment—and 74.3 ± 9.5 in the group B—patients with involved sustentacular fragment—with a significant difference between the two groups (*p* < 0.001). The present study was the first that compares clinical outcome in these two groups of fractures—intact vs involved sustentaculum—and reported that patients with fractured sustentaculum fragment were prone to develop worse outcomes than patients who had such fragment intact.

The Sanders classification, which focuses on STJ involvement, does not underline the importance of the CCJ [23]. However, in recent years there has been an increased interest on the reduction of CCJ. Gallino et al. [24] demonstrated that patients with fractures involving greater than 50% of the CCJ developed arthritic changes approximately 2 years after injury. We reported that the greater was the fragmentation of the posterior STJ according to Sanders classification, the higher was the CCJ fracture incidence. Our data were comparable with Vosoughi et al. [25] that found a correlation between Sanders-type fracture and the probability of CCJ involvement.

The present study had several limitations. It was a nonrandomized and retrospective single-center analysis of a relatively small cohort of patients. The merely clinical and not radiographic evaluation of patients in the postoperative phase had to be considered as another limitation. Despite these drawbacks, the present study is one of the few that investigated the clinical outcome of calcaneal fractures in regard to sustentaculum tali involvement. Future studies could measure the width of the sustentacular fragment in relation to the width of the healthy contralateral calcaneus by means of CT imaging on both feet. Performing CT studies to obtain measurements on healthy feet is difficult to justify ethically from a radiation safety perspective. Nevertheless, a new volumetric imaging technique called cone beam CT has emerged in recent years. It has been shown to provide excellent image quality for visualization of bone structure (0.075 to 0.4-mm isotropic voxels) at a truly low radiation dose compared with multi-slice CT [26]. The effective dose of the cone beam CT for foot and ankle examinations ranges from 0.9 to 14.3 μSv [27]. Furthermore, the relationship between the stability of the sustentacular fragment and the quality of reduction and fixation of fractures should be further investigated in the next future. The literature shows conflicting results both on outcomes of calcaneus fractures and the potential superiority of one treatment option over another or nonoperative treatment in complex calcaneal fractures such as Sanders type 3 and 4 fractures [22], without focusing on the involvement of the sustentaculum tali. Future researches will be necessary to investigate whether the involvement of the sustentaculum tali in the context of complex fractures may play a key role in driving treatment decisions toward either surgical or nonoperative strategies.

## Conclusions

Calcaneus fractures associated with sustentaculum tali involvement are rare injuries, usually caused by high energy mechanisms. CT imaging is mandatory to develop an appropriate surgical planning. Sustentaculum tali is involved in around one-third of calcaneal fractures, thus contradicting its supposed anatomic constant position. Our sample proved that Sanders type 3 and 4 calcaneus fractures were associated with sustentaculum tali and/or CCJ involvement more often than simpler ones. Furthermore, sustentaculum tali involvement is associated with significantly worse postoperative clinical outcomes underlying the crucial role of such fragment on the clinical course.
